# Antibacterial mechanism and *in vivo* efficacy of cladribine against carbapenem-resistant *Klebsiella pneumoniae*

**DOI:** 10.1186/s12866-026-05197-z

**Published:** 2026-05-22

**Authors:** Kai-Di Liu, Fan-Yue Wang, Jun-Qi Liu, Wei-Hua Hao, Li-Li Guo, Yong-Da Zhao, Xiao-Ping Liao, Min-Ge Wang

**Affiliations:** 1 Laboratory of Veterinary Pharmacology, College of Veterinary Medicine, South China Agricultural University, Guangzhou, China; 2 College of Agriculture and Biology, Liaocheng University, Liaocheng, China; 3 College of Veterinary Medicine, Qingdao Agricultural University, Qingdao, China

**Keywords:** CRKP, Cladribine, Antibacterial mechanism, Pulmonary infection, Therapeutic potential

## Abstract

**Background:**

The proliferation and dissemination of carbapenem-resistant *Klebsiella pneumoniae* (CRKP) constitutes a critical public health concern, underscoring the urgent need for novel antimicrobial agents. In this study, we identified cladribine, a synthetic purine analogue, as a potential antimicrobial candidate against CRKP.

**Results:**

Cladribine exhibited an MIC of 64 μg/mL against clinical CRKP isolates. Consistently, growth curve and time-kill assays also demonstrated that cladribine displays measurable inhibitory and bactericidal effects against CRKP, achieving a 4 log_10_ CFU reduction. Mechanistically, cladribine increased bacterial membrane permeability and induced oxidative stress, as evidenced by elevated intracellular reactive oxygen species (ROS) and malondialdehyde (MDA) levels, accompanied by the activation of oxidative stress-related genes. Cladribine also impaired bacterial energy metabolism, resulting in reduced succinate dehydrogenase (SDH) and pyruvate kinase (PK) activities and decreased ATP levels. In a mouse lung infection model, treatment with 10 mg/kg and 20 mg/kg cladribine significantly reduced pulmonary bacterial burden by approximately by 2.0 log_10_ CFU and 3.0 log_10_ CFU, respectively. In addition, cladribine alleviated CRKP-induced lung injury, and suppressed the transcription of pro-inflammatory cytokines, including TNF-α, IL-1β, and IL-6, indicating both antibacterial and anti-inflammatory effects in vivo.

**Conclusion:**

These findings suggest that cladribine has potential as a therapeutic candidate against CRKP infection and provide preliminary mechanistic insights that may contribute to a better understanding of its antibacterial activity.

**Supplementary Information:**

The online version contains supplementary material available at 10.1186/s12866-026-05197-z.

## Background

Antimicrobial resistance (AMR) has emerged as a critical threat to global public health, with multidrug-resistant (MDR) pathogens increasingly undermining the efficacy of existing antibiotic therapies [1, 2]. Among Gram-negative pathogens, *Klebsiella pneumoniae* has gained recognition as a significant global healthcare burden, accounting for one-third of Gram-negative bacterial infections in clinical settings [3, 4]. *K. pneumoniae* is not only a major opportunistic human pathogen but also a primary cause of infections in hospitalized individuals worldwide, frequently associated with pneumonia, wound infections, soft tissue infections, and urinary tract infections [5]. *K. pneumoniae* is also the leading cause of neonatal sepsis, ranking among the top three pathogens responsible for such cases in most regions [6, 7]. Alarmingly, up to 91% of infections have been linked to *K. pneumoniae* in Asia [8]. This concerning prevalence has led to *K. pneumoniae* being included on the WHO’s priority pathogen list [9, 10].

The alarming rise of carbapenem-resistant *Klebsiella pneumoniae* (CRKP), a high-priority MDR pathogen that has evolved resistance to last-resort antibiotics such as carbapenems, has further exacerbated the threat posed by *K. pneumoniae* infections [11]. CRKP strains are particularly concerning due to their rapid global dissemination, limited treatment options, and association with severe healthcare-associated infections and high mortality rates [12]. In China, CRKP was initially detected in Zhejiang Province in 2004, and has since evolved into a nationwide endemic threat, accounting for approximately 64% of carbapenem-resistant Enterobacteriaceae infections [13, 14]. In 2017, the World Health Organization designated CRKP as a critical-priority pathogen. Recent epidemiological studies have further identified CRKP as one of the most clinically significant multidrug-resistant Enterobacteriaceae, with a global prevalence rate of 5.43% [15]. Moreover, the multidrug-resistant nature of CRKP significantly prolongs hospitalization duration and elevates mortality risk to 70% in infected patients [16]. In this context, non-antibiotic compounds have garnered increasing attention due to their structural diversity, lower propensity and multifaceted bioactivities to induce resistance compared to conventional antibiotics. Given the rapid spread of CRKP and diminishing treatment options, identifying effective non-antibiotic therapeutic approaches has emerged as an urgent research imperative.

Cladribine is a purine nucleoside analog widely used in clinical medicine for therapeutic purposes [17]. In the treatment of multiple sclerosis (MS), cladribine demonstrates significant efficacy. Its primary mechanism of action involves selectively targeting lymphocytes (B cells and T cells), inducing immune cell apoptosis by interfering with DNA synthesis, thereby reducing autoimmune attacks on the central nervous system [18]. Cladribine also inhibits DNA repair enzymes (such as adenosine deaminase), leading to cancer cell apoptosis, thereby treating certain hematologic malignancies, including hairy cell leukemia and chronic lymphocytic leukemia [19]. Notably, while the immunomodulatory and antineoplastic mechanisms of cladribine have been well-elucidated in eukaryotic systems, its potential antibacterial properties, particularly against CRKP, remain an unexplored frontier.

This study was designed to systematically evaluate the antibacterial efficacy of cladribine against CRKP through both in vitro and in vivo experiments. Additionally, the study provides preliminary insights into the molecular mechanisms underlying antibacterial action of cladribine. These findings provide a foundation for future mechanistic investigations and suggest that cladribine may have potential for further exploration as an antibacterial agent against CRKP infections.

## Results

### In vitro antibacterial activity of cladribine

**Fig. 1:**
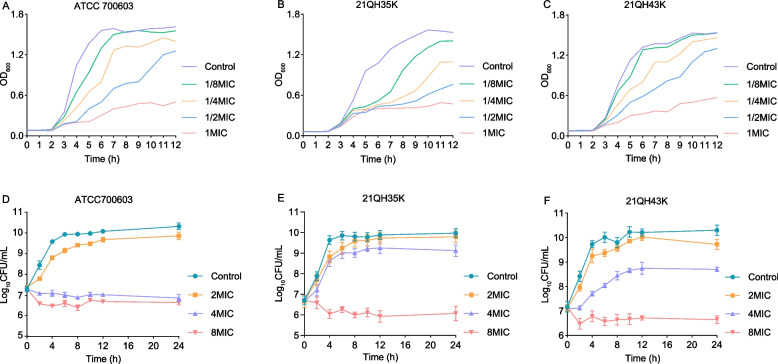
The antimicrobial and bactericidal activity of cladribine against *K. pneumoniae*. **A**-**C** Growth curve of cladribine against ATCC700603, 21QH35K and 21QH43K. **D**-**F** Time-kill curves cladribine against ATCC700603, 21QH35K and 21QH43K. Data are presented as mean ± SD from three independent experiments (*n* = 3)

Firstly, the antimicrobial potential of cladribine against CRKP isolates was assessed using the standardized broth microdilution assays, and it was found that cladribine exhibited antibacterial properties against both ATCC 700603 and clinical CRKP isolateswith a consistent minimum inhibitory concentration (MIC) of 64 μg/mL. The final concentration of DMSO used in the MIC assays was 2%, and control experiments confirmed that this concentration of DMSO did not affect bacterial growth (Fig S1). Then, the growth and time-kill curves were employed to further investigate the antibacterial activity of cladribine. The results demonstrated the growth of the CRKP isolates was inhibited by cladribine in a concentration-dependent manner (Fig. 1A-C). Meanwhile, time-kill curves showed that cladribine showed a concentration-dependent bactericidal effect against ATCC 700603 (Fig. 1D), 21QH35K (Fig. 1E) and 21QH43K (Fig. 1F), with significant reductions in bacterial counts observed at 8 × MIC, while lower concentrations (2 × MIC and 4 × MIC) mainly showed bacteriostatic activity. Collectively, these findings indicate that cladribine displays concentration-dependent antibacterial activity against CRKP isolates.

### Effect of cladribine on cell membrane integrity

**Fig. 2:**
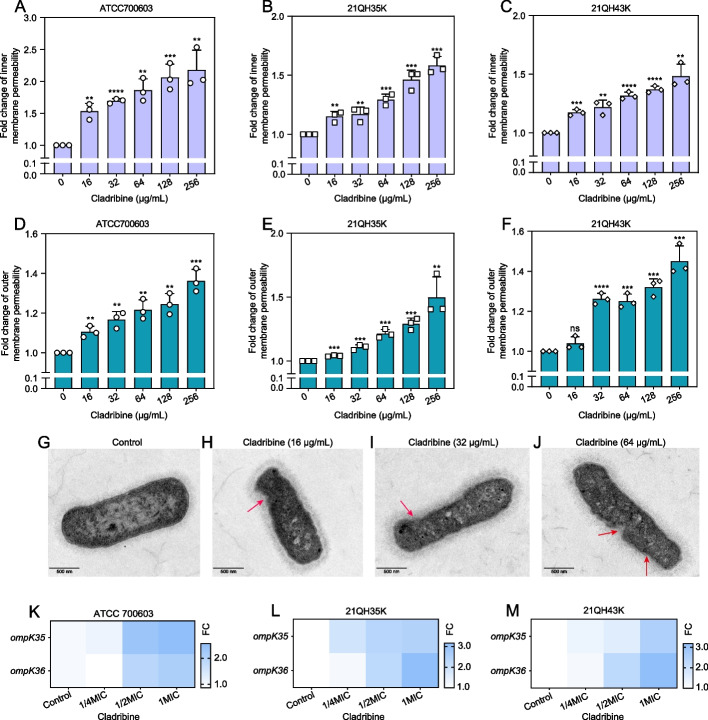
Cladribine increases cell membrane permeability. **A**-**C** Fold changes in inner membrane permeability. **D**-**F** Fold changes in outer membrane permeability. **G**-**J** Transmission electron microscopy (TEM) images of CRKP isolates from the control group and the cladribine-treated group. Red arrows indicate areas of blurred or disrupted bacterial cell boundaries. **K**-**M** Fold changes in relative expression levels of cell membrane-related genes. Quantitative data are expressed as mean ± SD from three independent experiments (*n* = 3). Statistical analysis was performed using one-way ANOVA. ns *P* > 0.05, **P < 0.01, ***P < 0.001, ****P < 0.0001

The leakage of intracellular protein and nucleic acid is widely recognized as a biomarker for assessing bacterial cell membrane integrity [20]. As shown in Fig S2, intracellular protein and nucleic acid leakage were significantly increased following the treatment of 16 ~ 256 μg/mL cladribine, suggesting leakage of intracellular macromolecules. To further evaluate the impact of cladribine on cellular membranes, membrane permeability was examined. The results demonstrated that inner membrane permeability exhibited a concentration-dependent increase following cladribine treatment, with significantly enhancements by 1.53-2.18folds for 700,603 (Fig. 2A), 1.18-1.58 folds for 21QH35K (Fig. 2B), and 1.17-1.48 folds for 21QH43K (Fig. 2C). Notably, cladribine also induced a similar increasing trend in outer membrane permeability (Fig. 2D-F). Moreover, TEM photomicrographs indicated showed morphological alterations of the cell envelope, including blurred cell boundaries, which became more evident at higher cladribine concentrations (Fig. 2G-J).

To further elucidate the antimicrobial mechanism of cladribine, transcriptional analysis of outer membrane genes *ompK35* and *ompK36* in CRKP was conducted following treatment with cladribine. RT-PCR analysis revealed that the expression of *ompK35 *and *ompK36* gene was upregulated to different extents under cladribine pressure (Fig. 2K-M, Fig S3A-C), suggesting its involvement in the bacterial response to cladribine-induced outer membrane stress. These results indicate that cladribine induces increased membrane permeability and membrane perturbation, which may play a role in its antibacterial activity.

### Cladribine induces oxidative stress response in bacteria

**Fig. 3:**
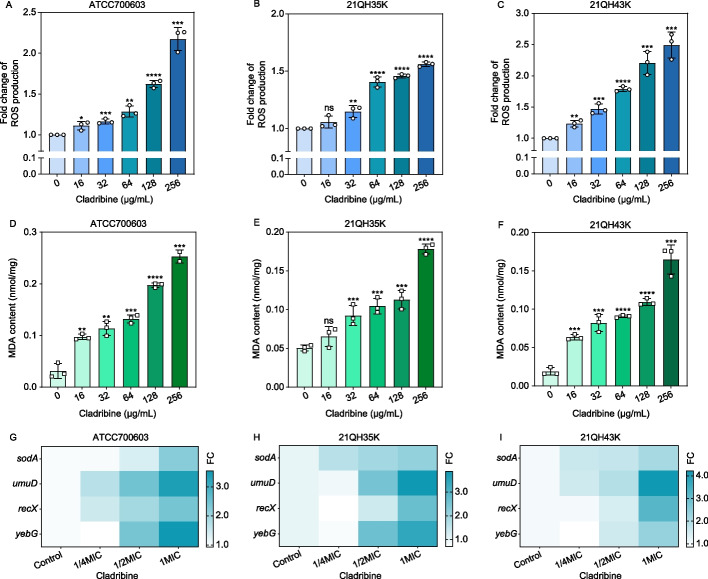
Cladribine induces the oxidative stress and SOS response. **A**-**C** Fold changes in ROS production. **D**-**F** MDA content. **G**-**I** Fold changes in relative mRNA expression levels of ROS and SOS response-related mRNA genes. All data are presented as mean ± SD from three independent experiments (n = 3). Data are presented as mean ± SD from three independent experiments (n = 3). Statistical significance was determined by one-way ANOVA. ns *P* > 0.05, *P < 0.05, **P < 0.01, ***P < 0.001, ****P < 0.0001

Oxidative stress has been implicated as a key contributor to bacterial cell damage and death [21]. Therefore, intracellular ROS levels and MDA content were assessed to characterize cladribine-induced oxidative stress in CRKP isolates. As shown in Fig. 3, cladribine significantly enhanced ROS accumulation in a concentration-dependent manner, with increases of 1.12-2.17 folds in ATCC700603(Fig. 3A), 1.24-2.49 folds in 21QH43K (Fig. 3B), and 1.15-1.56 folds in 21QH35K (Fig. 3C). The MDA levels as a marker of lipid peroxidation were also significantly elevated upon exposure to 16 ~ 256 μg/mL cladribine compared with control group (Fig. 3D-F). These results suggest that cladribine induces oxidative stress, which may contribute to its antibacterial activity. Moreover, the transcription levels of genes associated with antioxidant responses (*sodA*) and SOS response (*umuD*, *recX* and *yebG*) were further analyzed. The relative expression of gene coding superoxide dismutase (*sodA*) was noticeably upregulated under cladribine pressure compared with the control group. Meanwhile, key genes involved in the bacterial SOS response, including *umuD*, *recX* and *yebG*, were significantly upregulated following cladribine exposure (Fig. 3G-H, Fig S3D-F). These data demonstrate that cladribine induces oxidative stress and activates SOS-related gene expression, suggesting that oxidative damage may be involved in its antibacterial effects.

### Cladribine reduces the bacterial energy metabolism

**Fig. 4:**
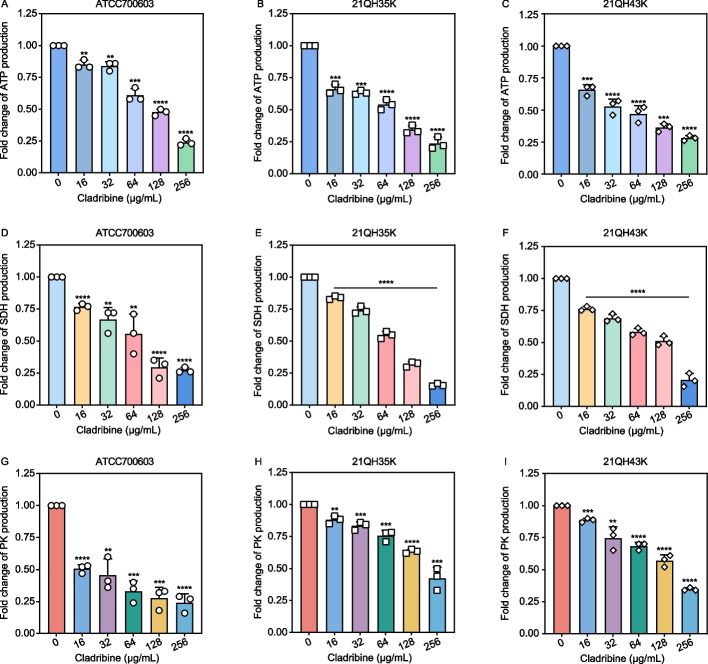
Cladribine reduces the bacterial energy metabolism. **A**-**C** Fold changes in ATP production. **D**-**F** Fold changes in SDH production. **G**-**I** Fold changes in PK production. Results are shown as mean ± SD from three independent experiments (*n* = 3). Statistical comparisons were conducted using one-way ANOVA. **P < 0.01, ***P < 0.001, ****P < 0.0001

The ATP levels, along with the activities of succinate dehydrogenase (SDH) and pyruvate kinase (PK) were further measured to evaluate the effects of cladribine on the energy metabolism of CRKP. The ATP levels was significantly suppressed by treatment with varying concentrations of cladribine (Fig. 4A-C). SDH and PK, two key enzymes involved in cellular energy metabolism, serve as indicators of metabolic activity. As shown in Fig. 4, cladribine reduced SDH activity in a dose-dependent manner in ATCC700603 (Fig. 4D), 21QH43K (Fig. 4E), and 21QH35K (Fig. 4F). A similar dose-dependent decline was observed in PK activity (Fig. 4G-I). These findings suggest that decreased SDH and PK activities may be associated with the reduced ATP levels observed. Taken together, the results indicate that cladribine affects bacterial energy metabolism, which may contribute to its antibacterial activity against CRKP.

### The antibacterial activity of cladribine against CRKP in vivo

To comprehensively evaluate the biosafety of cladribine, a series of in vitro and in vivo toxicity assessments were conducted, including hemolysis testing, cytotoxicity analysis, and acute toxicity studies in mice. In vitro safety profiling indicated cladribine caused no appreciable hemolysis of 2% sheep red blood cells over 0 ~ 512 µg/mL (Fig S4A). Consistently, CCK-8 assays showed comparatively low cytotoxicity in mammalian cells: the IC₅₀ for LX-2 cells was 2064 µg/mL (Fig S3B), whereas NRK-52E cells exhibited an IC_50_ of 509 µg/mL (Fig S3C). Notably, cell viability remained 95 ~ 100% in liver cells and 85 ~ 90% in kidney cells at 1 × MIC cladribine, indicating only mild effects at therapeutically relevant concentrations. In an acute toxicity study (10 ~ 20 mg/kg), liver and kidney histology revealed no overt lesions compared with controls (Fig S3D). Collectively, these findings demonstrate that cladribine shows acceptable in vitro and preliminary in vivo safety under the tested conditions.

**Fig. 5:**
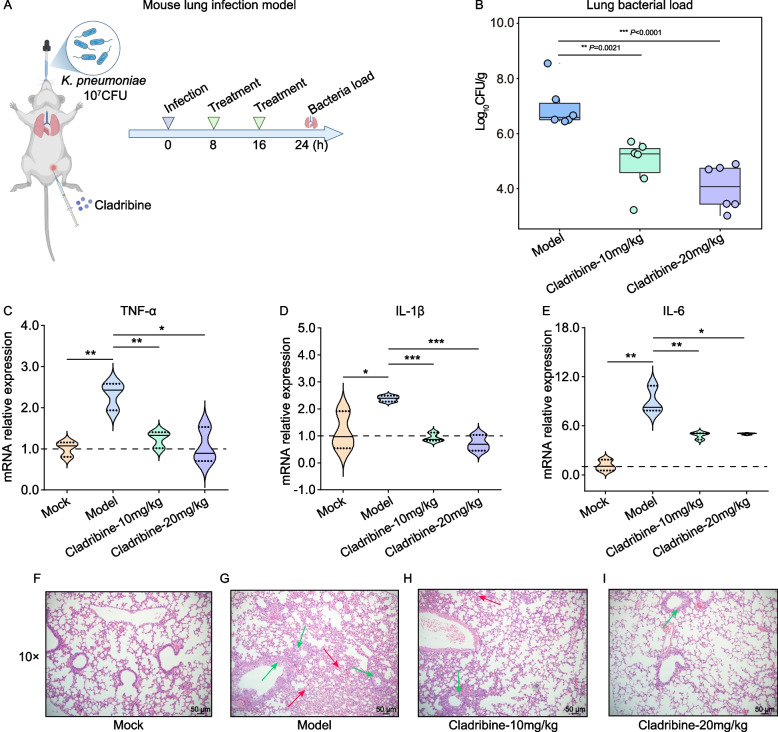
Therapeutic effects of cladribine in a murine pulmonary infection model induced by CRKP. **A** Experimental protocol for the mouse lung infection model. **B** The bacteria load in mouse lungs at 24 h post-infection. **C**-**E** Proinflammatory cytokine levels (TNF-α, IL-1β, IL-6) in lung tissue. **F**-**I** Hematoxylin and Eosin (H&E) staining of lung from mice at 24 h post-infection (Scar bar, 50 μm; Green arrow-Inflammatory cell infiltration; Red arrow-Decreased alveolar spaces). Data are presented as mean ± SD (n = 6 mice per group). Statistical analysis was performed using one-way ANOVA and independent-sample t test. *P < 0.05, **P < 0.01, ***P < 0.001

Subsequently, the in vivo therapeutic potential of cladribine was further evaluated in a murine lung infection model established using the clinical strain 21QH35K (Fig. 5A). The results showed that treatment with cladribine at doses of 10 mg/kg and 20 mg/kg reduced the pulmonary bacterial burden from 6.99 log_10_ CFU/g to 4.90 log_10_ CFU/g (*P* = 0.0021) and 4.40 log_10_ CFU/g (*P* < 0.0001), respectively (Fig. 5B). The effects on host inflammatory responses and pulmonary histopathology subsequently assessed following cladribine administration to further characterize the in vivo therapeutic potential of cladribine. Immunological analysis revealed that the expression levels of proinflammatory cytokines (TNF-α, IL-1β and IL-6) in the lung of infected mice was significantly reduced under cladribine treatment (*P* < 0.05) (Fig. 5C-E). In addition, histopathological analysis demonstrated the model group exhibited significant bronchial hyperplasia, extensive inflammatory cell infiltration, and disappearance of alveolar spaces in lung tissues compared with the control group. After treatment with cladribine at 10 mg/kg and 20 mg/kg, the severity of these pathological lesions gradually decreased (Fig. 5F-I). These observations suggest that cladribine reduces pulmonary bacterial burden and mitigates infection-associated inflammation in a murine CRKP pneumonia model, supporting its potential for further investigation as an antibacterial agent.

## Discussion

Carbapenem-resistant *Klebsiella pneumoniae* (CRKP) has emerged as a critical global public health threat, associated with high morbidity and mortality rates due to limited therapeutic options [22, 23]. The rapid dissemination of CRKP strains, coupled with their ability to acquire resistance mechanisms such as carbapenemase production (e.g., KPC, NDM, and OXA-48), has severely compromised the efficacy of last-resort antibiotics, including carbapenems and colistin [24, 25]. This underscores an urgent and unmet need to develop novel antimicrobial agents or alternative therapeutic strategies to effectively combat CRKP infections. In this study, we demonstrate that cladribine, a purine nucleoside analogue previously used in oncology and immunomodulatory therapy, exhibits measurable antibacterial activity against CRKP both in vitro and in vivo. Based on the established criteria for evaluating the antibacterial activity of pure compounds (MIC < 10 μg/mL indicates strong activity, 10 ~ 100 μg/mL moderate, and > 100 μg/mL weak or no activity) [26, 27], the observed MIC of 64 μg/mL suggested that cladribine possesses moderate antibacterial activity. While such activity may limit its feasibility as a standalone antibacterial monotherapy, it remains acceptable within the context of drug repurposing and early-stage antibacterial discovery. Growth curve and time-kill kinetics assay further supported the inhibitory effect of cladribine against CRKP and its rapid bactericidal activity. Previous studies have demonstrated that nucleoside analogs can disrupt DNA replication or induce metabolic stress in bacterial cells, contributing to their antimicrobial effects [28]. Importantly, the observed in vitro efficacy of cladribine against CRKP provides preliminary evidence supporting further mechanistic investigation. From a translational perspective, cladribine may be better positioned as a lead compound for further optimization or as an adjunct agent in combination therapy, rather than as a direct clinical antibiotic. Given the urgent need for alternative strategies against carbapenem-resistant pathogens, these findings support continued exploration of cladribine within a drug repurposing framework.

Cladribine induced a series of stress responses, including disruption of cell membrane integrity, induction of oxidative stress, and impairment of energy metabolism, which collectively compromised bacterial viability. It should be noted that, based on the current data, these effects are best interpreted as interconnected stress responses rather than fully established primary antibacterial targets. Disruption of cell membrane integrity represents an important and early antibacterial stress, as membrane perturbation can lead to increased permeability, leakage of intracellular components, and destabilization of cellular homeostasis [29, 30]. In this study, cladribine induced leakage of intracellular proteins and nucleic acids from CRKP isolates, indicating compromised membrane integrity. Consistently, a marked increase in membrane permeability and observable alterations in membrane morphology to the bacterial cell envelope were observed upon cladribine treatment. The transcriptional upregulation of *ompK35* and *ompK36* likely reflects a bacterial adaptive response to membrane stress rather than direct targeting of porins, highlighting that membrane perturbation is a key early event but may not represent the sole bactericidal mechanism. Previous studies have also shown that modulation of porin expression can influence bacterial susceptibility by altering membrane permeability and facilitating antibiotic influx or efflux [31]. Collectively, these observations suggest that membrane perturbation is a key component of cladribine-induced stress, but it is unlikely to represent the sole or isolated mechanism of antibacterial activity.

Oxidative stress is another prominent response observed following cladribine exposure. Excessive intracellular accumulation of reactive oxygen species (ROS) is known to exacerbate cellular damage and is often considered a downstream or amplifying factor contributing to bacterial cell death [32, 33]. In this study, cladribine induced a pronounced oxidative stress response, as evidenced by elevated intracellular ROS and MDA levels and activation of antioxidant and SOS-response genes. These changes are consistent with activation of stress-responsive pathways and may reflect a compensatory response to cladribine-induced oxidative stress [34, 35]. However, the present data are correlative, and whether ROS generation is a primary driver of bacterial killing or a downstream consequence of membrane and metabolic disruption remains to be determined. Future studies employing ROS scavengers or genetic mutants deficient in oxidative stress pathways would be valuable to clarify causality.

Disruption of bacterial energy metabolism is a critical aspect of antibacterial action [36, 37]. In the present study, cladribine treatment led to a significant reduction in intracellular ATP levels in CRKP isolates, suggesting an adverse effect on the bacterial energy production machinery. To further elucidate the underlying mechanisms, we examined the activities of key metabolic enzymes involved in central carbon metabolism. Succinate dehydrogenase (SDH) and pyruvate kinase (PK) were selected as representative enzymes of bacterial energy metabolism, as SDH plays a central role in linking the tricarboxylic acid cycle with the respiratory chain, whereas PK is a key rate-limiting enzyme in glycolysis responsible for substrate-level ATP generation [38, 39]. Given the central role of membrane integrity in maintaining proton gradients and respiratory function, and the known sensitivity of metabolic enzymes to oxidative damage, it is plausible that membrane perturbation and ROS accumulation jointly contribute to metabolic collapse. Taken together, these findings support a model in which cladribine induces a network of interconnected stress responses, including membrane damage, oxidative stress, and metabolic impairment, which collectively undermine bacterial viability rather than acting through a single dominant primary target.

The safety evaluation of a candidate compound is a prerequisite for its further pharmacological development. In the present study, cladribine exhibited acceptable in vitro and preliminary in vivo safety within the tested dose range, as evidenced by the absence of significant hemolysis, low cytotoxicity toward hepatic and renal cells, and the lack of obvious histopathological abnormalities in major organs. These findings supported the feasibility of evaluating its in vivo antibacterial efficacy. Notably, the observed therapeutic benefit in vivo likely reflects a combined effect of reduced bacterial load and modulation of infection-associated inflammatory responses, rather than a single dominant mechanism. Collectively, these findings indicated that cladribine shows potential for further exploration in the context of CRKP infection models.

Several limitations of this study should be acknowledged. First, the antibacterial activity of cladribine was evaluated using a limited panel of CRKP isolates, which may not fully capture the genetic and phenotypic diversity of clinical CRKP populations. Second, the mechanistic insights presented here are primarily based on phenotypic, biochemical, and transcriptional correlations, and direct causal relationships between individual pathways and bacterial killing were not established. Third, in vivo efficacy was assessed without comparison to a positive antibiotic control, and pharmacokinetic, and long-term safety studies were not performed. These limitations highlight the need for broader strain testing, mechanistic validation, and advanced in vivo evaluation in future studies. Despite these limitations, this work provides initial evidence that cladribine exerts antibacterial activity against CRKP through a multifaceted stress-inducing mechanism and demonstrates its therapeutic potential in a murine pneumonia model.

## Conclusion

This study showed that cladribine possesses antibacterial activity against CRKP in both in vitro assays and a murine lung infection model. Mechanistic investigations revealed that cladribine treatment was associated with increased membrane permeability, elevated oxidative stress markers, and alterations in bacterial energy metabolism, suggesting that multiple stress-related pathways may contribute to its antibacterial activity. In vivo, cladribine reduced pulmonary bacterial burden and was associated with decreased inflammatory cytokine expression and attenuated infection-related lung injury. These findings support further investigation of cladribine as a potential antibacterial candidate against CRKP and provide preliminary insights into its possible modes of action.

## Methods

### Bacterial isolates, compound and antibiotics

The strains used in this study included *Klebsiella pneumoniae* ATCC 700603 and clinical CRKP isolates 21QH35K, 21QH43K, GD18-KP-60, and VH1-2. Specifically, ATCC 700603 was originally obtained from the American Type Culture Collection (ATCC, Virginia, USA) and preserved in our laboratory. Two CRKP isolates (21QH35K and 21QH43K) were recovered from fecal samples of migratory birds in 2021 and carried the *bla*_KPC-2_ gene [40]. In addition, two CRKP isolates harboring *bla*_KPC-2_ were obtained in 2018 from a patient throat swab (GD18-KP-602) and a vegetable sample (VH1-2), respectively. The isolates have been preserved in our laboratory and their use in this study was approved and authorized by the Academic Committee of South China Agricultural University. Detailed characteristics of these strains are provided in Table S1. All bacterial strains were preserved in LB broth containing 30% glycerol and stored in cryovials at −80 °C. Cladribine (purity > 98%) was obtained from MedChemExpress (Shanghai, China), while the antibiotics were procured from McLin Bio-Chemical Technology (Shanghai, China). Stock solutions were prepared by dissolving cladribine in dimethyl sulfoxide (DMSO) and antibiotics in sterile distilled water, respectively.

### Minimum inhibitory concentration (MIC) determination

The minimal inhibitory concentrations (MICs) of cladribine against CRKP isolates were determined using the broth microdilution method in accordance with the Clinical and Laboratory Standards Institute guidelines (CLSI) [41]. Briefly, two-fold serial dilutions of cladribine were prepared in Mueller–Hinton broth to a final volume of 100 μL in 96-well microtiter plates. Bacterial cultures in the exponential growth phase (1^10^8^ CFU/mL) were diluted 1:100 with MH broth, followed by the addition of 100 μL diluted culture per well. The MIC results were interpreted after incubation at 37 °C for 16–18 h.

### Growth curve assays

Growth curves assay was performed to evaluate the effect of cladribine on bacterial growth as previously described [42]. Overnight bacterial cultures were diluted to an OD_600_ of 0.1 in fresh LB broth. Cladribine was then added to the cultures at various concentrations, and growth curves were generated by monitoring the absorbance at 600 nm of bacterial cultures over a 12 h period. The experiment was conducted in triplicate to ensure biological reproducibility.

### Time-kill assays

Overnight cultures were adjusted to an initial inoculum of 1 × 10^7 CFU/mL in fresh Mueller–Hinton (MH) broth. Subsequently, 10 mL bacterial suspension were transferred to sterile 50 mL conical tubes and treated with cladribine at various concentrations. The control group without cladribine treatment. At designated time points (0, 2, 4, 6, 8, 10, 12 and 24 h), 100 µL samples collected from each tube, serially diluted in PBS, and plated on LB agar. Colony counts were determined at each time point after incubation at 37 °C for 18–24 h. Time-kill curves were generated by plotting log10 CFU/mL versus time to evaluate the bactericidal kinetics of cladribine.

### Evaluation of the cell membrane permeability

The permeability of inner membrane and outer membrane were assessed using propidium iodide (PI) and N-phenyl-1-naphthylamine (NPN), respectively. Briefly, overnight bacterial cultures were resuspended in PBS to an OD_600_ of 0.5. The resuspended bacteria were then incubated with various concentrations of cladribine at 37 °C for 2 h, followed by incubation with 10 μM PI or NPN in the dark for 30 min. Then, the samples were centrifuged and washed twice with PBS to remove any unbound probe. Fluorescence intensity was measured using an excitation wavelength of 535 nm and an emission wavelength of 615 nm for PI, and an excitation wavelength of 350 nm with an emission wavelength of 420 nm for NPN. All experiments were conducted in triplicate to ensure reproducibility. In additions, membrane permeability was assessed using transmission electron microscopy (TEM) to observe any potential alterations in cell membrane integrity following exposure to cladribine.

### Leakage of protein and nucleic acid

The bacteria were cultured to the exponential-growth phase, washed and resuspended in PBS solution to a concentration of' 1x10^8 CFU/mL. Cladribine was then added to the suspension to achieve final concentrations of 1/4 MIC, 1/2 MIC, 1 MIC, 2 MIC, and 4 MIC. Following incubation at 37 °C for 12 h, the bacterial cultures were centrifuged at 5000 rpm for 10 min at 4 °C. The supernatant was aspirated and filtered through a 0.22 μm membrane. Finally, the absorbance of the filtrate at 260 nm and 280 nm was measured using a UV-1801 ultraviolet spectrophotometer (Beijing, China) to assess the leakage of nucleic acids and proteins, respectively.

### Measurement of ROS production and MDA level

Intracellular ROS production was measured using a cellular ROS detection assay kit (Sigma, China) according to the manufacturer's instructions. Briefly, bacteria were cultured overnight at 37 °C, then suspended in PBS to an OD_600_ of 0.5. The suspended bacteria were co-incubated with 2′,7′-Dichlorofluorescein diacetate (DCFH-DA) in the dark at 37 °C for 30 min. Subsequently, cladribine was added to the bacterial suspension and incubated for 2 h. The fluorescence intensity was then measured with excitation at 488 nm and emission at 525 nm. Fluorescence values were normalized to bacterial cell density (OD_600_). Malondialdehyde (MDA) level was assessed using the Malondialdehyde Content Assay Kit (Solarbio, China) following the manufacturer’s protocol. After treatment with various concentrations of cladribine, the bacterial cultures were lysed and centrifuged to collect the supernatant. The absorbance at 532 nm was then measur566ed using a microplate reader and values were normalized to bacterial cell density (OD_600_).

### Measurement of intracellular ATP and enzymes associated with energy production

Intracellular ATP concentrations were assessed using the Enhanced ATP Assay Kit (Beyotime, China). The enzyme activities of succinate dehydrogenase (SDH) and pyruvate kinase (PK) were measured by enzyme detection kits (Jiancheng, China) according to the manufacturers' protocols. Specifically, bacteria were cultured at 37 °C for 12 h and washed with PBS to an OD_600_ of 0.5. Bacterial suspensions were treated with varying concentrations of cladribine and incubated for 12 h. Afterward, the bacteria were harvested, resuspended in lysis buffer, and subjected to ultrasonic disruption. The supernatant was collected for subsequent assays of ATP content, SDH and PK activity. ATP concentrations and enzyme activity values were normalized to bacterial cell density (OD_600_).

### RNA extraction and RT-PCR analysis

Overnight bacterial cultures were inoculated into fresh LB broth supplemented with various concentrations of cladribine, and incubated at 37 °C for 4 h. Total RNA was then extracted using the OMEGA Total RNA Kit I (Omega, China). Reverse transcription was performed with the HiScript III RT SuperMix for qPCR (+ gDNA wiper) (Vazyme, China) to synthesize cDNA from the RNA. The cDNA was subsequently analyzed by RT-qPCR using the SYBR qPCR Master Mix Kit (Vazyme, China) and optimized primers (Table S2). The 16S rRNA gene was used as an internal control. The RT-qPCR reaction included four steps: initial pre-denaturation at 95 °C for 30 s, denaturation at 95 °C for 15 s (repeated for 40 cycles), annealing at 60 °C for 60 s, and extension at 72 °C for 30 s. All experiments were performed in triplicate.

### Safety evaluation

For in vitro cytotoxicity assessment, cladribine (0 ~ 512 μg/mL) were first tested for hemolytic activity using a 2% sheep blood cell suspension, which was purchased from Guangzhou Ruite Biotechnology Co., Ltd. (Guangzhou, China). The viability of human hepatic stellate cells LX-2 and rat renal tubular epithelial cells NRK-52E after 12 h exposure to cladribine (0 ~ 1024 μg/mL) was then evaluated with a CCK-8 assay (Vazyme, China). Among them, LX-2 cells and NRK-52E cells were purchased from Shanghai Fuheng Biotechnology Co., Ltd. (Shanghai, China) and the American Type Culture Collection (ATCC, Virginia, USA), respectively. Subsequently, absorbance at 450 nm was recorded using a microplate reader (PerkinElmer, USA), and cell viability was calculated according to the manufacturer’s protocol. For in vivo toxicity evaluation, mice received cladribine (10 or 20 mg/kg) once daily for three days before euthanasia. Subsequently, liver and kidney tissues were subjected to hematoxylin and eosin (H&E) staining. PBS-treated mice served as controls.

### Animal experiment

A mouse pneumonia model was established to evaluate the therapeutic effect of cladribine against CRKP in vivo. Specific pathogen-free (SPF) ICR female mice aged 5–6 weeks, weighing 18–22 g, were purchased from Zhuhai Bestest biological Technology Co., LTD. Mice were anesthetized via intraperitoneal injection of tribromoethanol before tracheal inoculation. Each mouse received a 100 µL suspension of (10^8 CFU/mL) bacteria, delivered dropwise into the trachea. Mice were randomly assigned to treatment and control groups (n = 6 per group) following bacterial inoculation. Treatment groups received cladribine at 10 mg/kg and 20 mg/kg via intraperitoneal injection, with the initial administration at 8 h post-infection followed by a second dose at 16 h. At 24 h post-infection, mice were humanely euthanized by cervical dislocation under anesthesia. The lungs were aseptically collected, homogenized in 1 mL sterile PBS using a tissue grinder, and serially diluted for quantitative bacterial culture on LB agar plates. Bacterial loads were expressed as log10 CFU per gram of lung tissue.

Lung tissue was harvested and immediately frozen in liquid nitrogen. Total RNA was extracted using TRIzol reagent (Vazyme, China) following the manufacturer’s protocol. cDNA was synthesized using a reverse transcription kit (Vazyme, China). RT-qPCR was performed with SYBR Green Master Mix (Vazyme, China) on a CFX96 system (Bio-Rad), targeting TNF-α, IL-1β and IL-6 (Table S2). β-actin served as the endogenous control. Data were analyzed via the 2^−ΔΔCt^ method. For comprehensive histopathological evaluation, left lung tissues from each experimental group were fixed in 2.5% paraformaldehyde (PFA) for 24 h, paraffin-embedded, and sectioned at 4 μm thickness. Tissue sections were then stained with hematoxylin and eosin (H&E) following standard protocols for microscopic assessment of inflammatory infiltration, alveolar architecture, and other pathological changes.

### Statistical analysis

GraphPad Prism 8.0 software and RStudio version 3.4.1 were used for statistical analysis and figure generation. The results were analyzed by analysis of variance (ANOVA) and independent-sample t test. The corrected *P* values of < 0.05 were considered to indicate statistical significance.

## Supplementary Information

Supplementary Material 1.

## Data Availability

All data generated or analyzed during this study are included in this article.
