# Supplementary material for: Antibacterial mechanism and in vivo efficacy of cladribine against carbapenem-resistant Klebsiella pneumoniae
Source: BMC Microbiol. 2026 May 22;26:626. doi: 10.1186/s12866-026-05197-z (PMC13371293; doi:10.1186/s12866-026-05197-z)
Supplement: Supplementary file 1 — Supplementary Material 1. [file 12866_2026_5197_MOESM1_ESM.docx]

**Supplementary material**

**Antibacterial mechanism and *in vivo* efficacy of cladribine against carbapenem-resistant *Klebsiella pneumoniae***

Kai-Di Liu^1^, Fan-Yue Wang^1^, Jun-Qi Liu^1^, Wei-Hua Hao^1^, Li-Li Guo^3^, Yong-Da Zhao^3^,

Xiao-Ping Liao^1^*, Min-Ge Wang^1,2^*

^1^ Laboratory of Veterinary Pharmacology, College of Veterinary Medicine, South China Agricultural University, Guangzhou, China

^2^ College of Agriculture and Biology, Liaocheng University, Liaocheng, China

^3^ College of Veterinary Medicine, Qingdao Agricultural University, Qingdao, China

***Corresponding author:** Xiao-Ping Liao, Email: [xpliao@scau.edu.cn](mailto:xpliao@scau.edu.cn); Min-Ge Wang, Email: [wangminge@lcu.edu.cn](mailto:wangminge@lcu.edu.cn).

**Table S1 Source information of *K. pneumoniae* used in this study.**

| Strain | Species | Origin | Isolation time | Carbapenem resistance genes | MIC (μg/ml) | | |  |
| --- | --- | --- | --- | --- | --- | --- | --- | --- |
|  |  |  |  |  | MEM | IMP | ERT | |
| ATCC 700603 | *K. pneumoniae* | Laboratory strain | - | - | 0.032 | 0.125 | 0.064 | |
| 21QH35K | CRKP | Migratory bird faeces | 2021 | KPC-2 | 64 | 16 | >64 | |
| 21QH43K | CRKP | Migratory bird faeces | 2021 | KPC-2 | 64 | 16 | >64 | |
| GD18-KP-602 | CRKP | Patient's throat swab | 2018 | KPC-2 | 8 | 2 | 32 | |
| VH1-2 | CRKP | Vegetable | 2018 | KPC-2 | 64 | 16 | >64 | |

MEM: Meropenem, IMP: Imipenem, ERT: Ertapenem.

**Table S2 RT-PCR primers used in this study.**

| Primer | Sequences (5’-3’) |
| --- | --- |
| *ompK35-F* | CTGCAAAATATGACGCCAACA |
| *ompK35-R* | GTTTTGCCAGCGAAGTAGTTACC |
| *ompK36-F* | GGTAGCAGGCGCAGCAA |
| *ompK36-R* | TCGCCTTTCACGCCTACAC |
| *sodA-F* | GAAAGGCGATAAACTGGCGG |
| *sodA-R* | GCGCCAGAAATAGCTTCACC |
| *umuD-F* | GCCCGACGGTACAGCTTATT |
| *umuD-R* | ACACCAAAGACATCCAGCGT |
| *recX-F* | GCAAAGGTTATGGACCTGCG |
| *recX-R* | GCATCGCTTTTTCTGTCGCT |
| *yebG-F* | CATTCGTGAGGGCGAAGAGA |
| *yebG-R* | GAAAGGGCTTCACGTTGCTC |
| 16S-F | ATTAGATACCCTGGTAGTCCACGC |
| 16S-R | TTGCGGGACTTAACCCAAC |
| IL-1β-F | GACCTTCCAGGATGAGGACA |
| IL-1β-R | AGGCCACAGGTATTTTGTCG |
| IL-6-F | CCGGACAGGAGACTTCACAG |
| IL-6-R | TCCACGATTTCCCAGAGAAC |
| TNF-α-F | CTCTTCAAGGGACAAGGCTG |
| TNF-α-R | CGGACTCCGCAAAGTCTAAG |
| β-actin-F | ACTGCCGCATCCTCTTCCT |
| β-actin-R | TCAACGTCACACTTCATGATGGA |


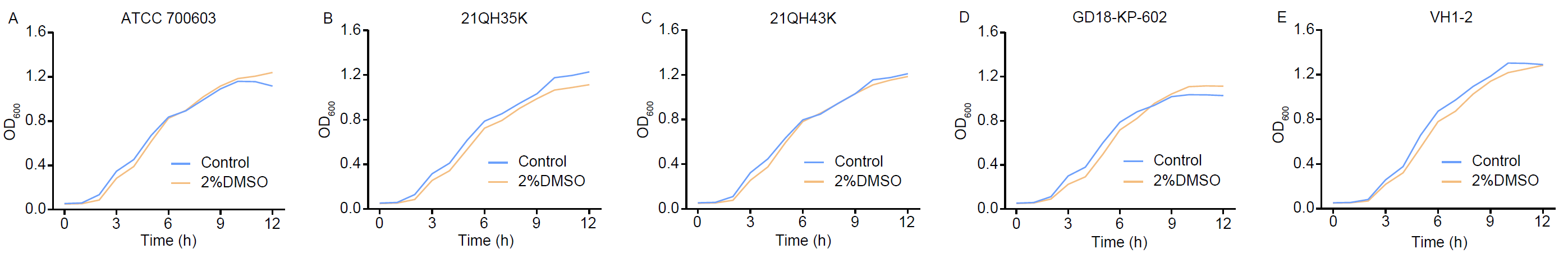


**Figure S1 Growth curves of *Klebsiella pneumoniae* strains in the presence or absence of 2% DMSO.**

Bacterial growth was monitored by measuring optical density at 600 nm (OD_600_) over a 12 h incubation period. (A) ATCC 700603; (B) 21QH35K; (C) 21QH43K; (D) GD18-KP-602; (E) VH1-2.


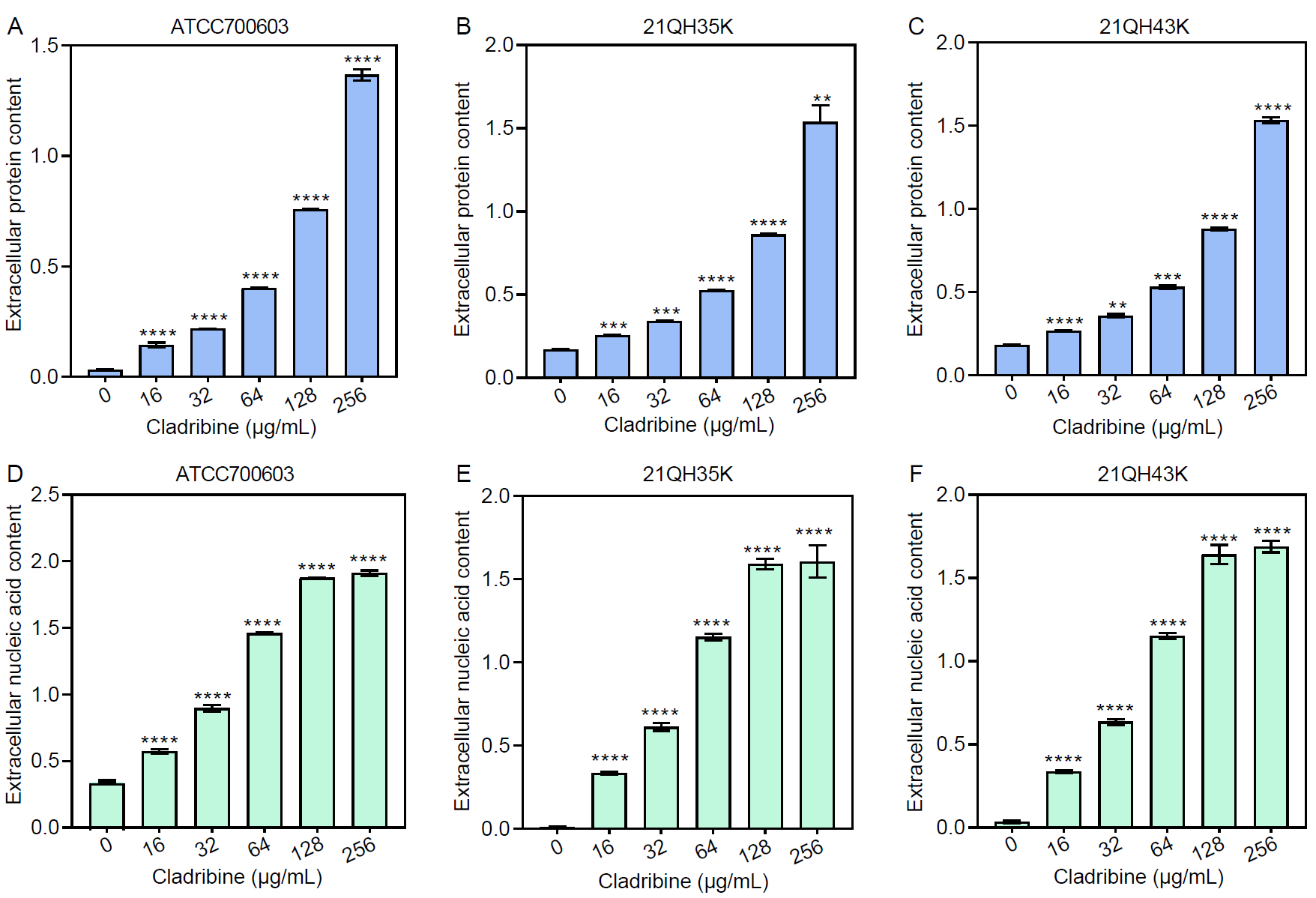


**Figure S2** **Leakage of intracellular material in bacteria.**

(A-C) Protein leakage. (D-F) Nucleic acid leakage. Data are expressed as mean ± SD from three independent experiments (n = 3). Statistical analysis was performed using one-way ANOVA. ***P* < 0.01, ****P* < 0.001, *****P* < 0.0001.


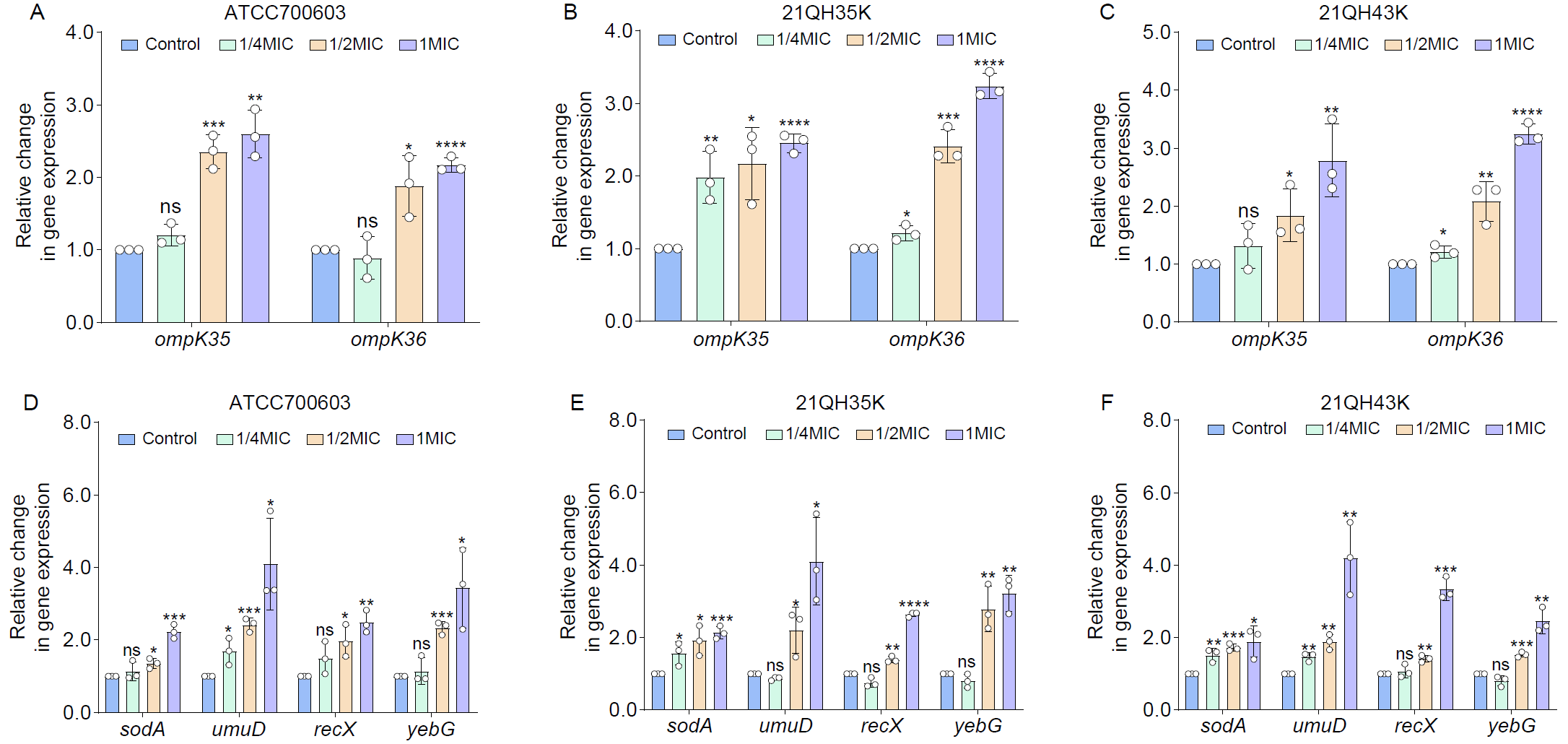


**Figure S3 mRNA expression levels of genes associated with outer membrane and antioxidant responses following cladribine exposure.**

(A-C) outer membrane genes. (D-F) antioxidant responses genes. Results are shown as mean ± SD from three independent experiments (n = 3). Statistical comparisons were conducted using one-way ANOVA. ns *P* > 0.05, **P* < 0.05, ***P* < 0.01, ****P* < 0.001, *****P* < 0.0001.


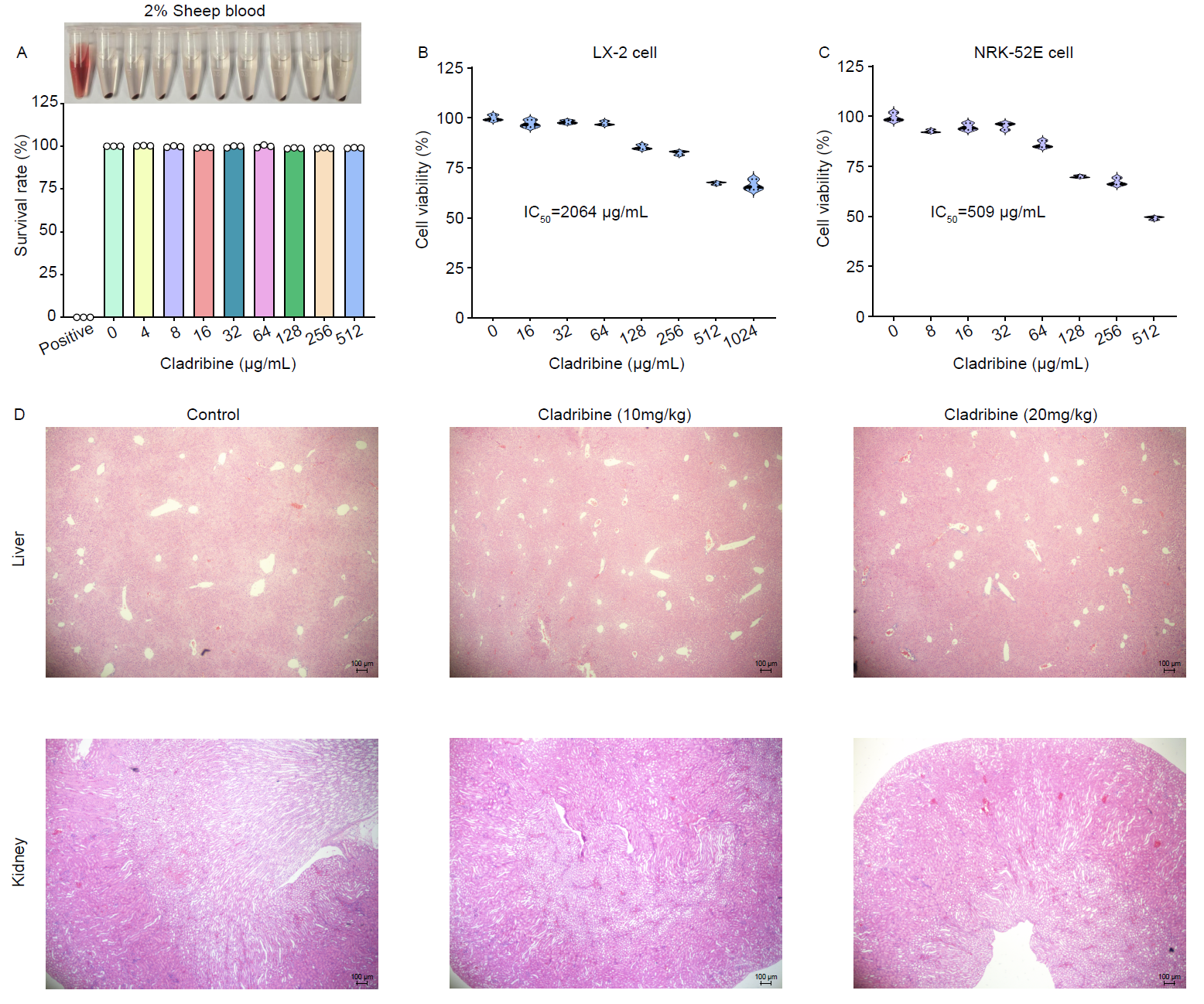


**Figure S4 Evaluation of the biosafety of cladribine.**

(A) Hemolysis assay of 2% sheep blood cells treated with cladribine. (B-C) Cell viability of liver cells LX-2 (B) and kidney cells NRK-52E (C) incubation with cladribine determined by the CCK-8 assay. (D) H&E staining of liver and kidney tissues from mice treated with cladribine (10 or 20 mg/kg) for three days (Scale bar = 100 μm). Data are presented as mean ± SD from three independent experiments (n = 3).
